# Supplementary material for: A Sensitive and Transparent Method for Tumor-Informed Detection of Circulating Tumor DNA in Ovarian Cancer Using Whole-Genome Sequencing
Source: Int J Mol Sci. 2024 Dec 12;25(24):13349. doi: 10.3390/ijms252413349 (PMC11678811; doi:10.3390/ijms252413349)
Supplement: Supplementary file 1 [file ijms-25-13349-s001.zip › Supplementary Material incl. Figures.pdf]

Supplementary Material

Supplementary Figures

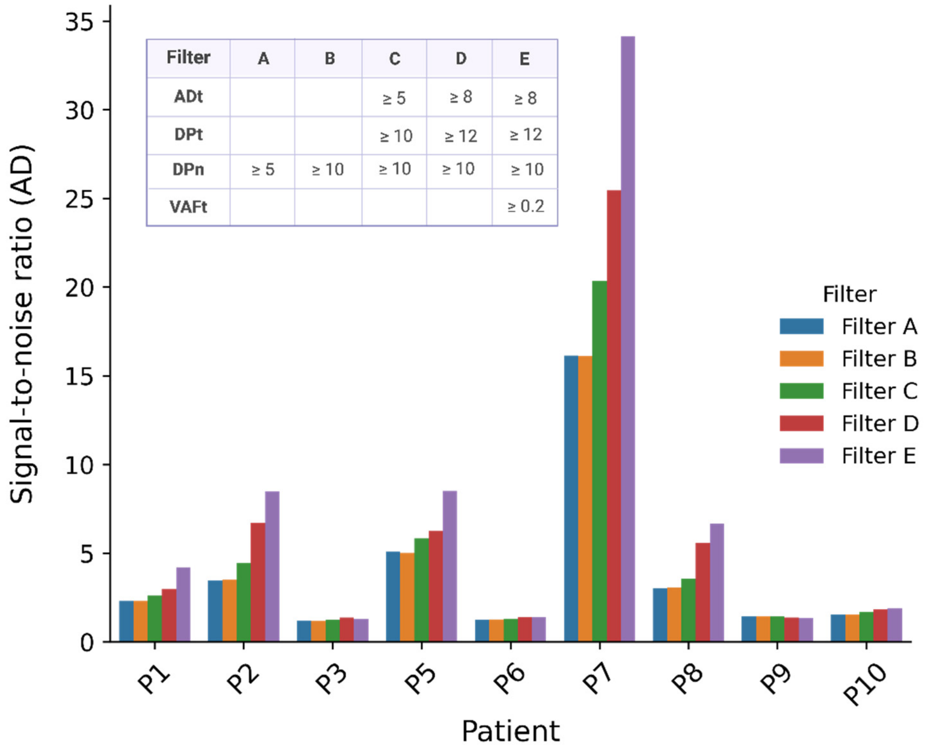

Figure S1. Overview of AD signal-to-noise ratios after applying different filters.
The stringency of the filter parameters increases progressively from Filter A to E as the filter threshold is altered for AD and DP of tumor and normal as well as, VAF of tumor. 1000_g PON and ADn = 0 are applied to all. ADt (allelic depth in tumor), DPt (read depth in tumor), VAFt (variant allele frequency in tumor), ADn (allelic depth in normal), DPn (read depth in normal).

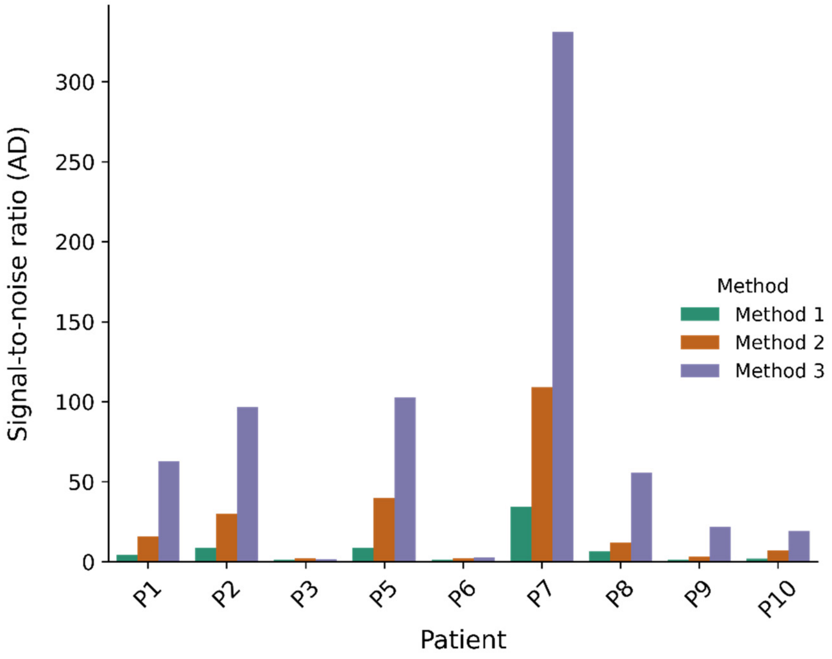

Figure S2. Overview of signal-to-noise ratios for AD when different methods for filtering noise are applied.

Applied filtering parameters: **Methods 1**: 1000_g PON, **Method 2:** merged PON, **Method 3**: merged PON and plasma pool filter

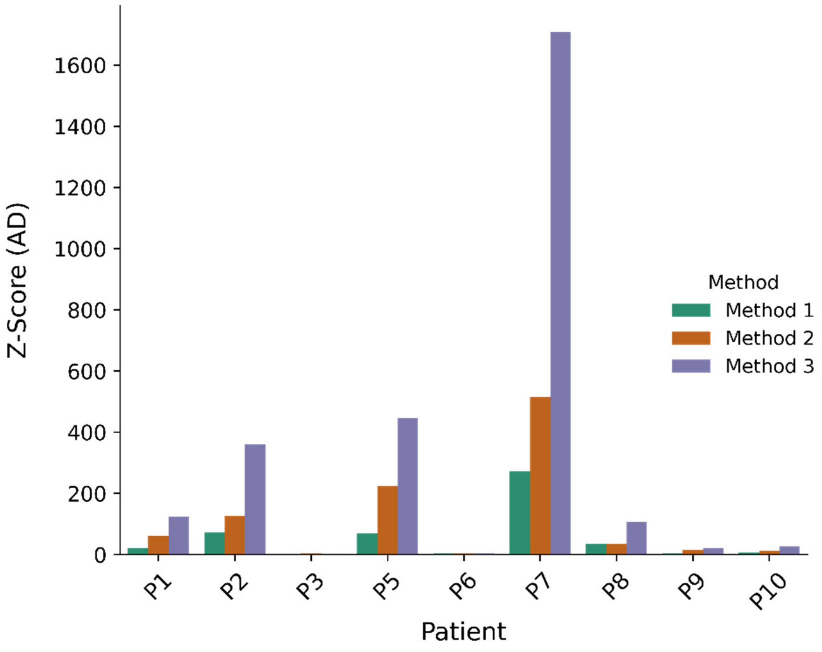

Figure S3. Overview of Z-Scores computed based on AD when different methods for filtering noise are applied.

Applied filtering parameters: **Method 1**: 1000_g PON, **Method 2**: merged PON, **Method 3**: merged PON and plasma pool filter. Filtering thresholds ADt ≥ 8; DPt ≥ 12; ADn=0; DPn ≥ 10; VAFt ≥ 0.2 are used in all three methods.

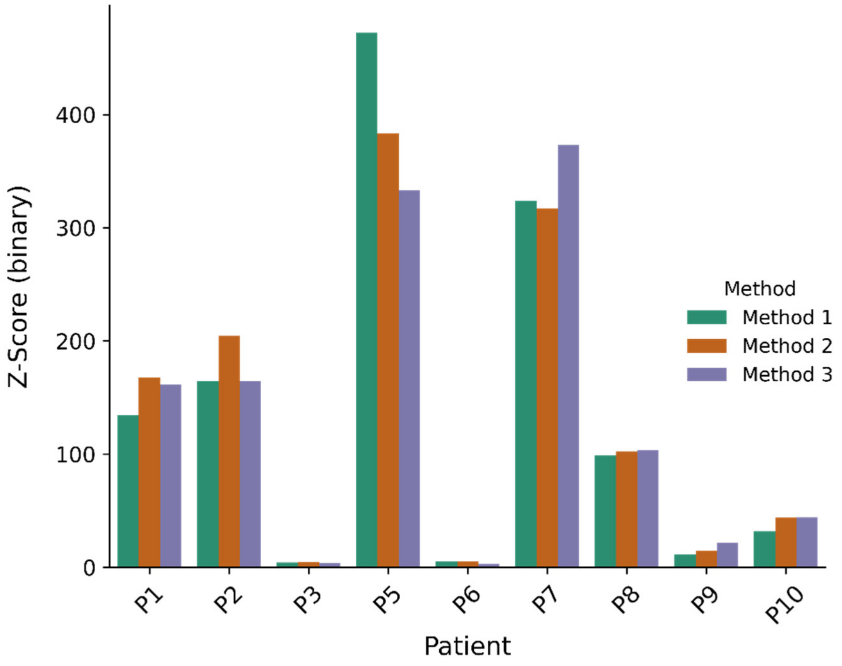

Figure S4. Overview of binary Z-Scores when different methods for filtering noise are applied.

Applied filtering parameters: **Method 1**: 1000_g PON, **Method 2:** merged PON, **Method 3**: merged PON and plasma pool filter. Filtering thresholds ADt ≥ 8; DPt ≥ 12; ADn=0; DPn ≥ 10; VAFt ≥ 0.2 are used in all three methods.

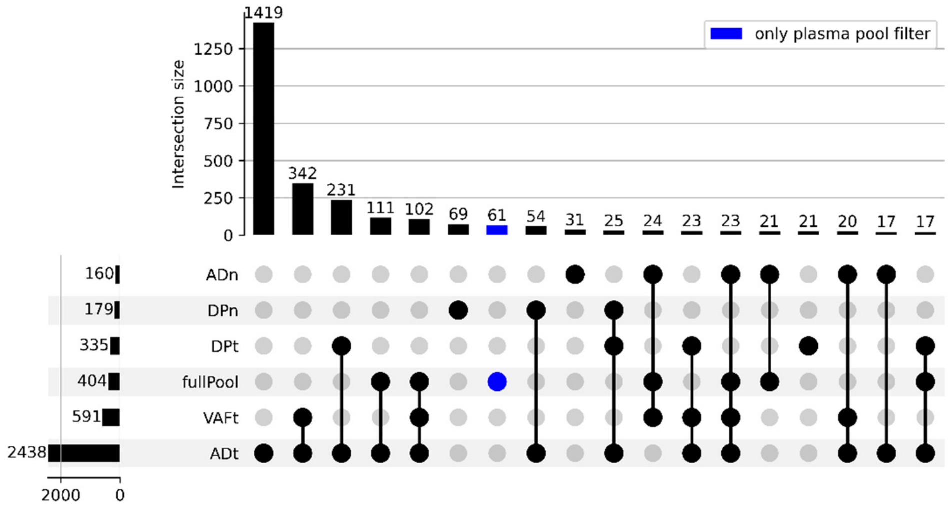

Figure S5. Overview of the number of variants removed by filters and the overlap between them for patient 2.

ADn (alternate allelic depth in normal), DPn (read depth in normal), DPt (read depth in tumor), fullPool (plasma pool filter), VAFt (variant allele frequency in tumor), ADt (alternate allelic depth in tumor).

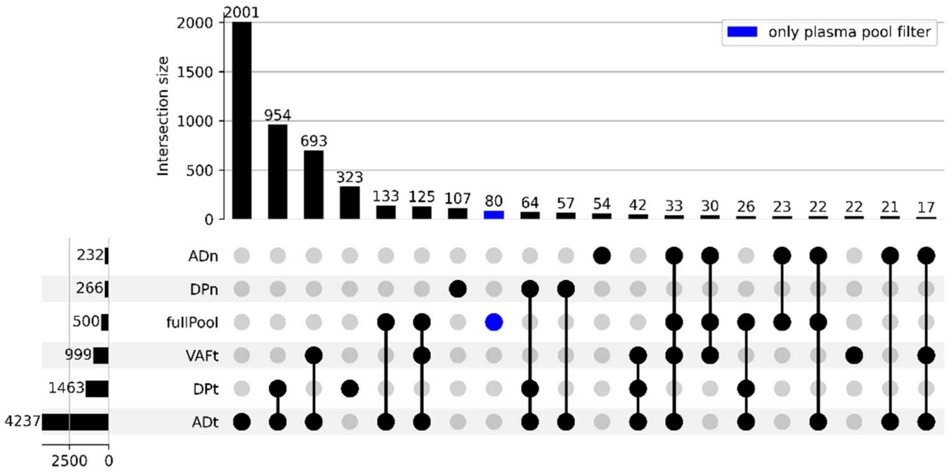

Figure S6. Overview of the number of variants removed by filters and the overlap between them for patient 3.

ADn (alternate allelic depth in normal), DPn (read depth in normal), fullPool (plasma pool filter), VAFt (variant allele frequency in tumor), DPt (read depth in tumor), ADt (alternate allelic depth in tumor).

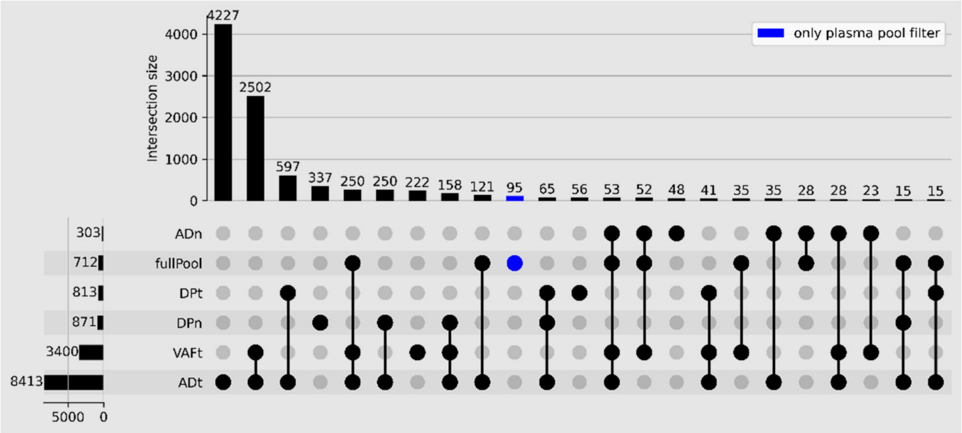

Figure S7. Overview of the number of variants removed by filters and the overlap between them for patient 5.

ADn (alternate allelic depth in normal), fullPool (plasma pool filter), DPt (read depth in tumor), DPn (read depth in normal), VAFt (variant allele frequency in tumor), ADt (alternate allelic depth in tumor).

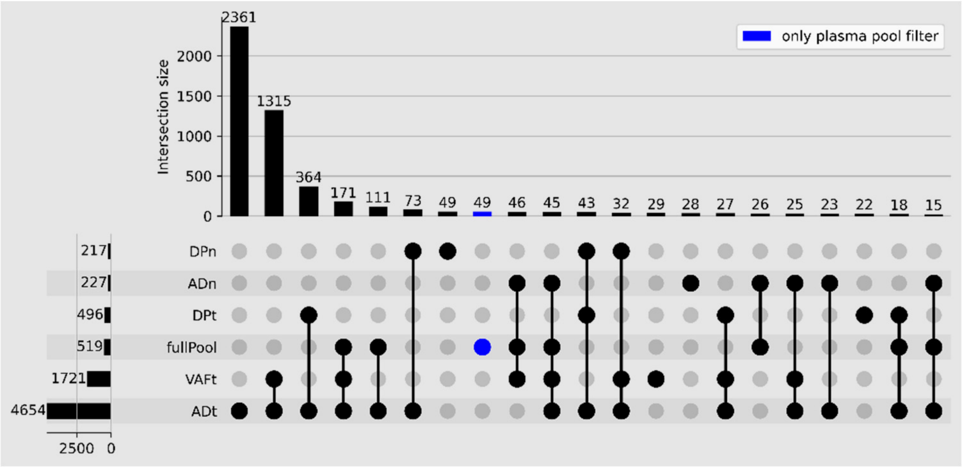

Figure S8. Overview of the number of variants removed by filters and the overlap between them for patient 6.

DPn (read depth in normal), ADn (alternate allelic depth in normal), DPt (read depth in tumor), fullPool (plasma pool filter), VAFt (variant allele frequency in tumor), ADt (alternate allelic depth in tumor).

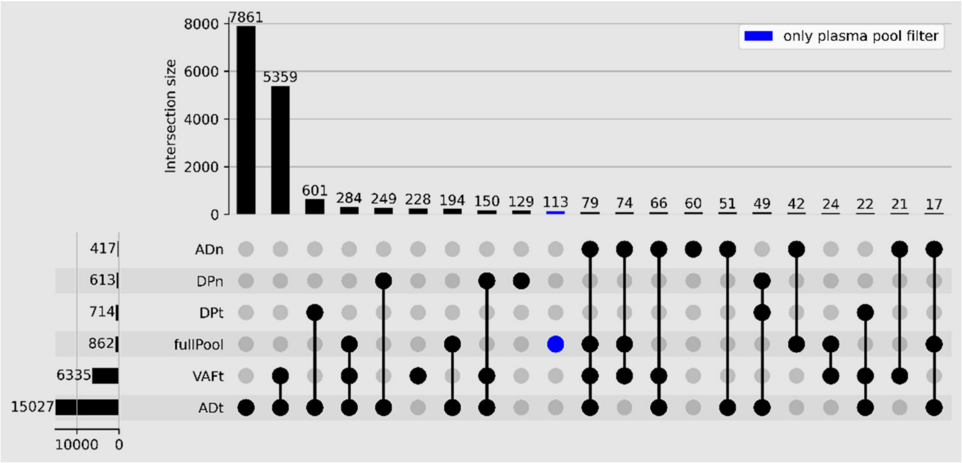

Figure S9. Overview of the number of variants removed by filters and the overlap between them for patient 7.

ADn (alternate allelic depth in normal), DPn (read depth in normal), DPt (read depth in tumor), fullPool (plasma pool filter), VAFt (variant allele frequency in tumor), ADt (alternate allelic depth in tumor).

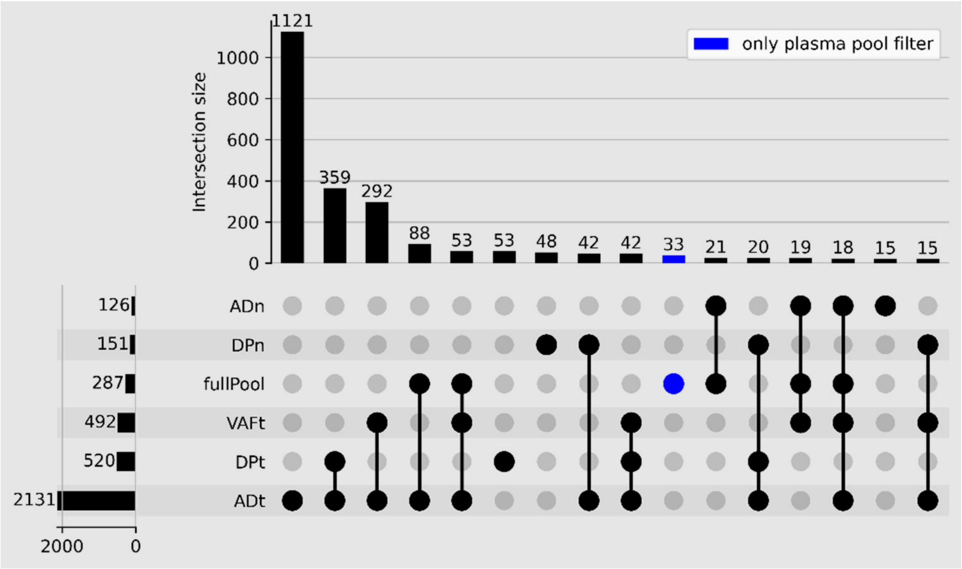

Figure S10. Overview of the number of variants removed by filters and the overlap between them for patient 8.

ADn (alternate allelic depth in normal), DPn (read depth in normal), fullPool (plasma pool filter), VAFt (variant allele frequency in tumor), DPt (read depth in tumor), ADt (alternate allelic depth in tumor).

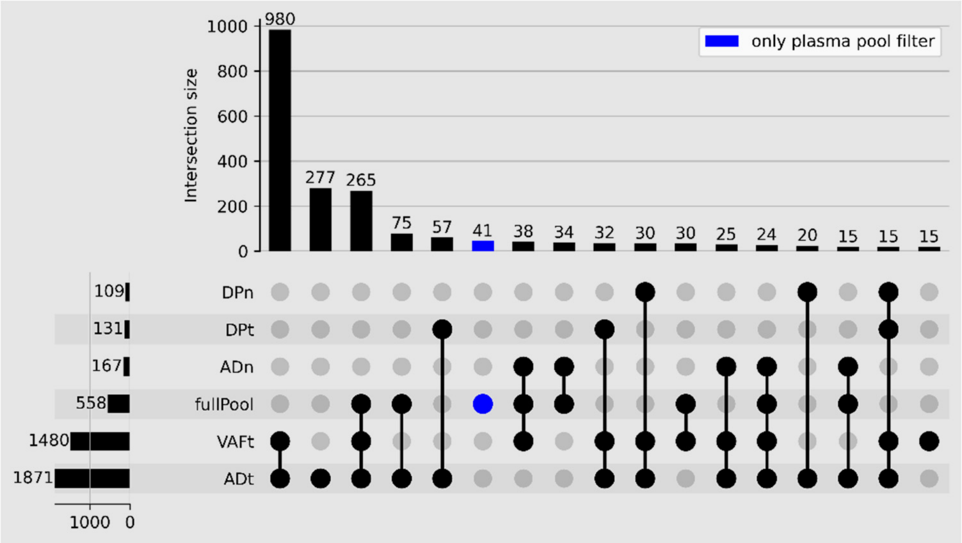

Figure S11. Overview of the number of variants removed by filters and the overlap between them for patient 9.

DPn (read depth in normal), DPt (read depth in tumor), ADn (alternate allelic depth in normal), fullPool (plasma pool filter), VAFt (variant allele frequency in tumor), ADt (alternate allelic depth in tumor).

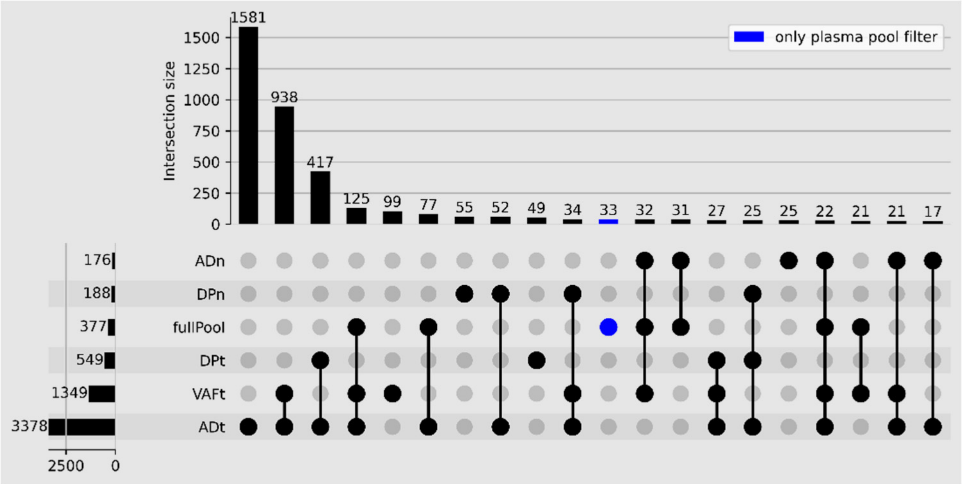

Figure S12. Overview of the number of variants removed by filters and the overlap between them for patient 10.

ADn (alternate allelic depth in normal), DPn (read depth in normal), fullPool (plasma pool filter), DPt (read depth in tumor), VAFt (variant allele frequency in tumor), ADt (alternate allelic depth in tumor).
